# ENDOCRINOLOGY IN THE TIME OF COVID-19: Remodelling diabetes services and emerging innovation

**DOI:** 10.1530/EJE-20-0377

**Published:** 2020-06-05

**Authors:** Deborah J Wake, Fraser W Gibb, Partha Kar, Brian Kennon, David C Klonoff, Gerry Rayman, Martin K Rutter, Chris Sainsbury, Robert K Semple

**Affiliations:** 1 Usher Institute, University of Edinburgh, Edinburgh, UK; 2 Edinburgh Centre for Endocrinology & Diabetes, NHS Lothian, Edinburgh, UK; 3 Portsmouth Hospital NHS Trust, Portsmouth, UK; 4 NHS Greater Glasgow and Clyde, Glasgow, UK; 5 Mills-Peninsula Medical Center, San Mateo, California, USA; 6 Ipswich Hospital, East Suffolk and North East Essex NHS Trust, Colchester, UK; 7 University of East Anglia, Norwich, UK; 8 Division of Diabetes, Endocrinology and Gastroenterology, School of Medical Sciences, University of Manchester, Manchester, UK; 9 Manchester Diabetes Centre, Manchester University NHS Foundation Trust, Manchester Academic Health Sciences Centre, Manchester, UK; 10 Institute of Applied Health Research, University of Birmingham, Birmingham, UK; 11 Centre for Cardiovascular Sciences, The Queens Medical Research Institute, University of Edinburgh, Edinburgh, UK

## Abstract

The COVID-19 pandemic is a major international emergency leading to unprecedented medical, economic and societal challenges. Countries around the globe are facing challenges with diabetes care and are similarly adapting care delivery, with local cultural nuances. People with diabetes suffer disproportionately from acute COVID-19 with higher rates of serious complications and death. In-patient services need specialist support to appropriately manage glycaemia in people with known and undiagnosed diabetes presenting with COVID-19. Due to the restrictions imposed by the pandemic, people with diabetes may suffer longer-term harm caused by inadequate clinical support and less frequent monitoring of their condition and diabetes-related complications. Outpatient management need to be reorganised to maintain remote advice and support services, focusing on proactive care for the highest risk, and using telehealth and digital services for consultations, self-management and remote monitoring, where appropriate. Stratification of patients for face-to-face or remote follow-up should be based on a balanced risk assessment. Public health and national organisations have generally responded rapidly with guidance on care management, but the pandemic has created a tension around prioritisation of communicable vs non-communicable disease. Resulting challenges in clinical decision-making are compounded by a reduced clinical workforce. For many years, increasing diabetes mellitus incidence has been mirrored by rising preventable morbidity and mortality due to complications, yet innovation in service delivery has been slow. While the current focus is on limiting the terrible harm caused by the pandemic, it is possible that a positive lasting legacy of COVID-19 might include accelerated innovation in chronic disease management.

## Foreword

This publication was created through collaborative working between UK and International diabetes leaders and experts. It represents the situation, as of 12 April 2020, 4 weeks post ‘lock-down’, at which time 10 621 people have died of COVID-19 in the UK (1) and 99 690 internationally (2). The current UK Government advice to people with diabetes is to follow general public ‘stay at home guidance’. Shielding/complete self-isolation is not currently stipulated for diabetes, unlike very high-risk individuals with severe respiratory illnesses or compromised immunity. This advice is largely mirrored internationally.

## Diabetes and COVID-19

COVID-19 has resulted in the biggest disruption to healthcare delivery in living memory. New policy and healthcare working practice have been rapidly introduced. This article focuses on changes to diabetes care delivery during the pandemic. Currently it is unclear whether people with diabetes are at higher risk of contracting COVID-19. However, they are clearly at higher risk of poor outcomes once infected. Among 7162 US cases reported by the CDC (28 March), the percentage of COVID-19 patients with at least one underlying health condition (e.g diabetes) was nearly three-fold higher among those requiring (1) intensive care unit admission (78%) and (2) hospitalisation (71%) compared to people not hospitalised (27%) (3, 4). People with diabetes may also have a ~2–3 fold increased mortality following COVID-19 infection in early reports (4, 5). Age, gender, multimorbidity, low socioeconomic status, and degree of pathogen exposure are risk factors for disease severity, but it is not yet clear which are independent contributors (5, 6, 7, 8, 9).

## Acute care for individual people with diabetes and COVID-19

**Figure 1:**
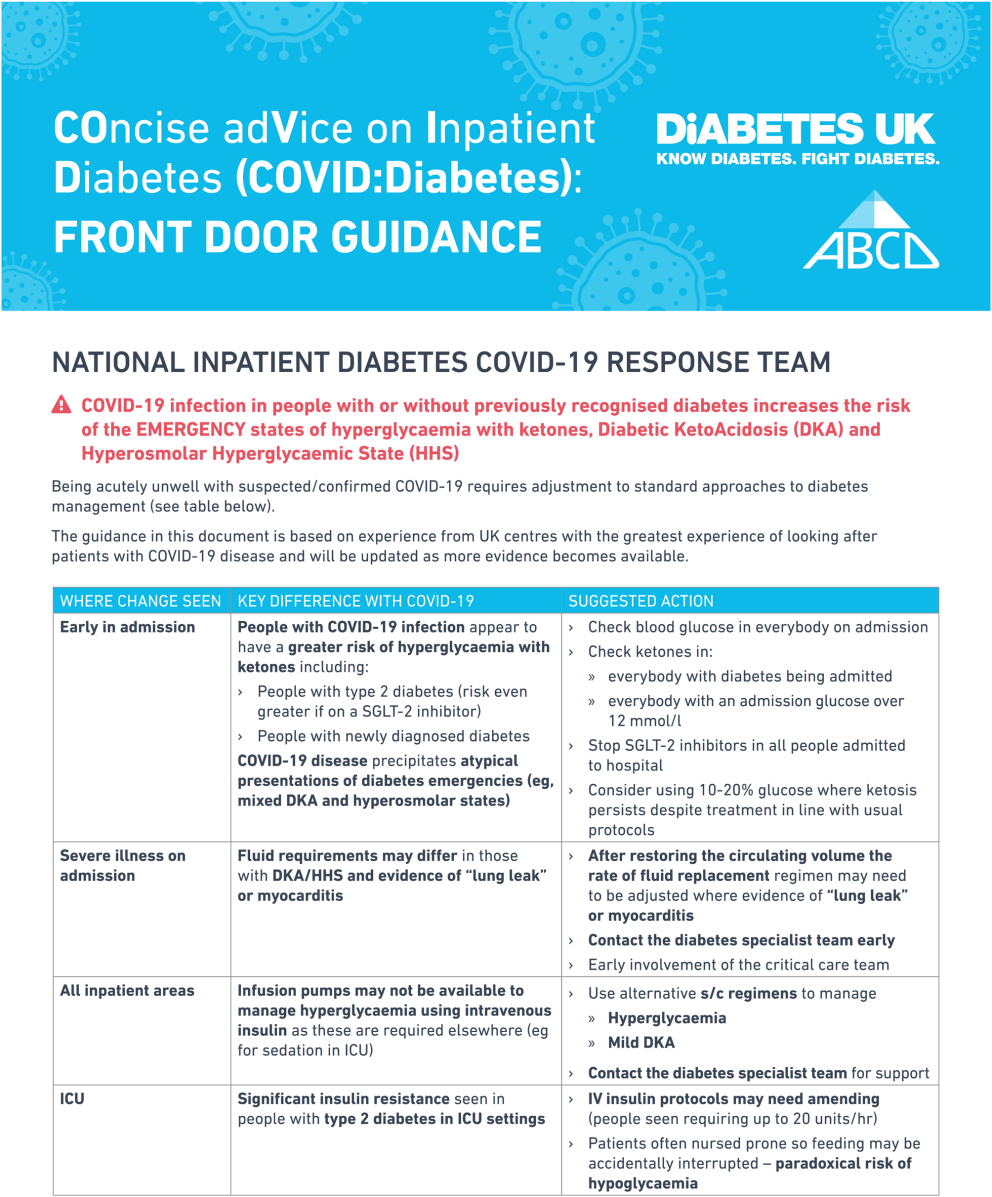

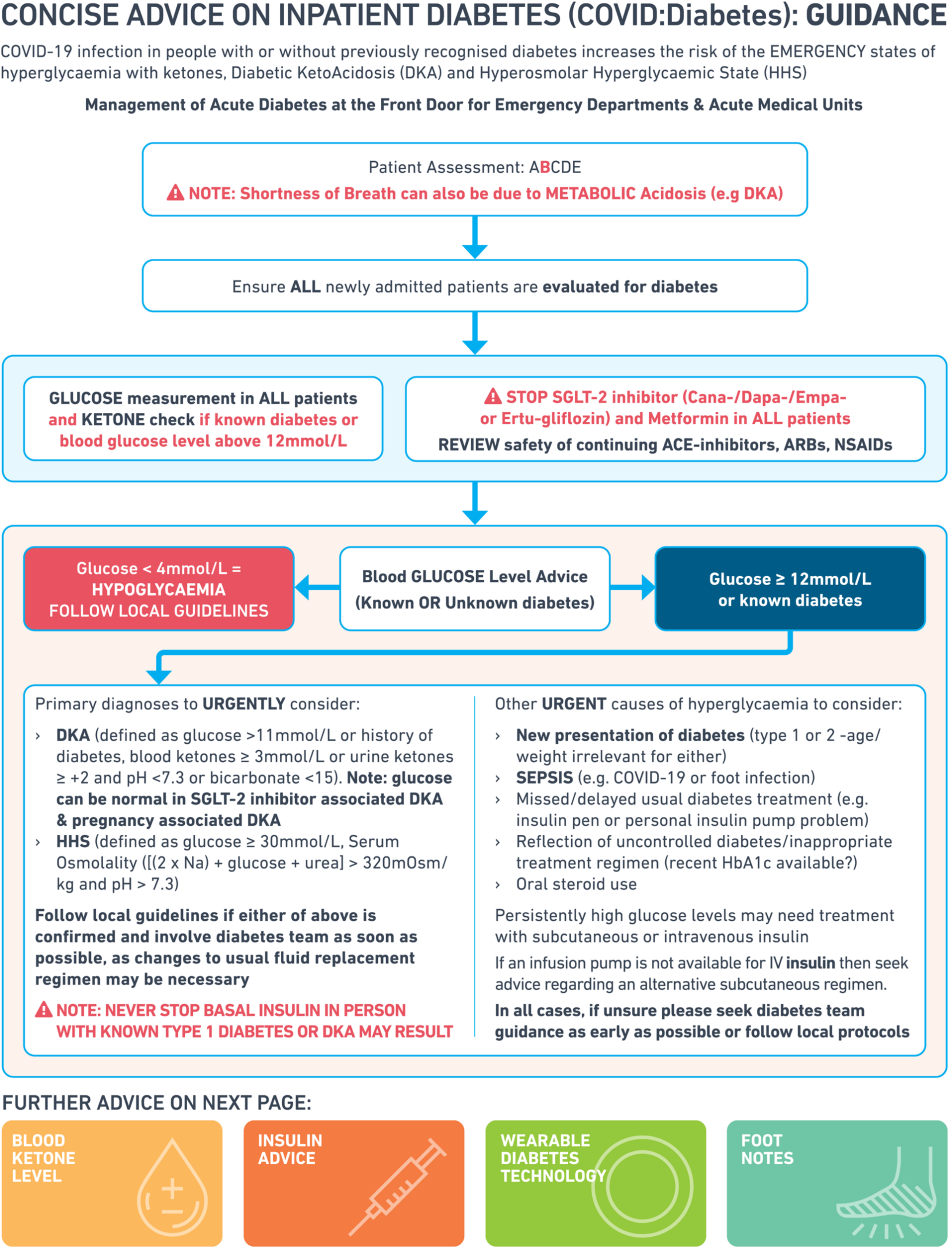

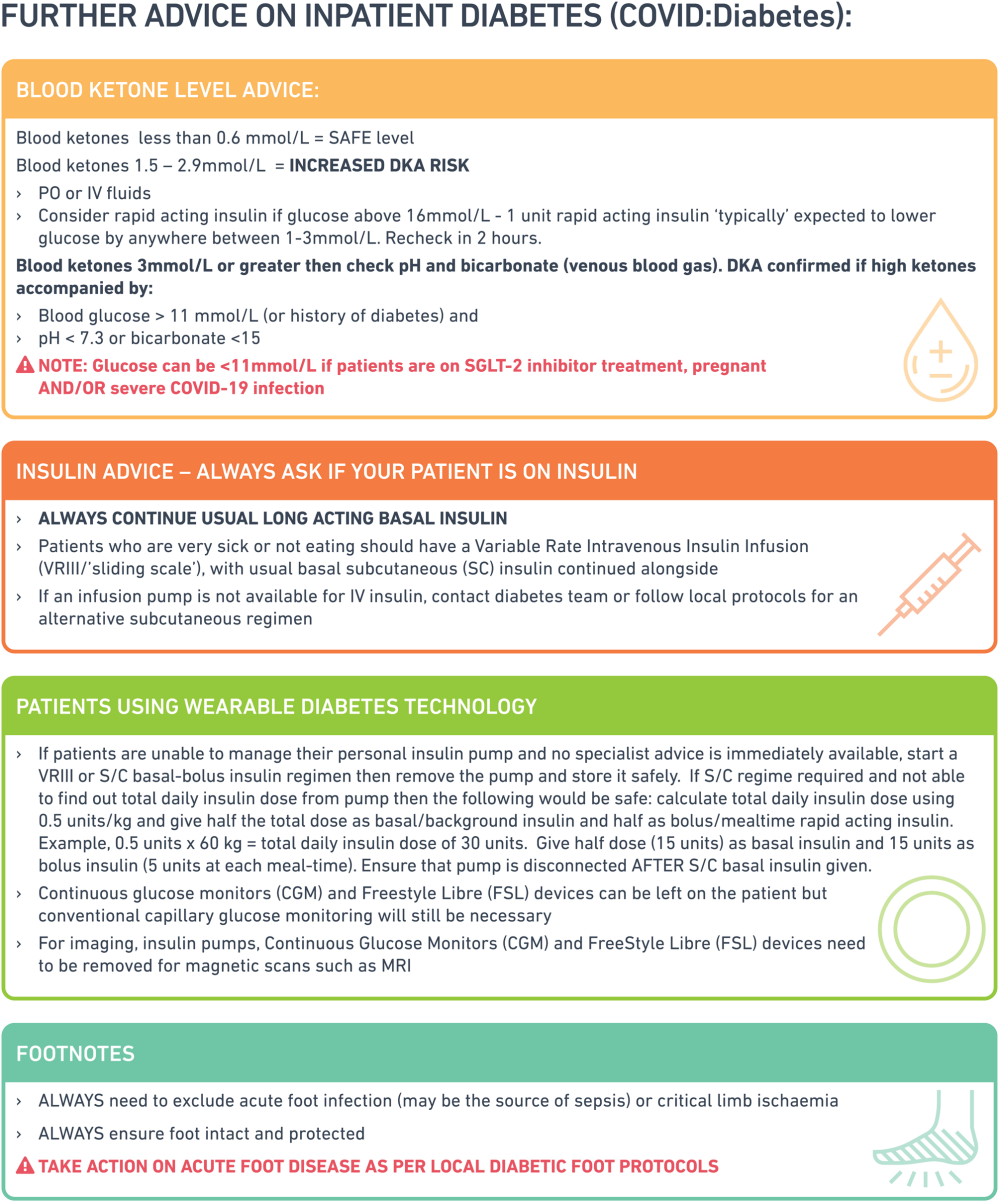
Represents a consensus document to support the management of inpatient diabetes during COVID-19 based on practice from a number of UK centres. Included with permission from lead authors.

Acute illness suspected or confirmed to be due to COVID-19 may require modification of current guidelines, particularly for safe use by staff unfamiliar with diabetes management, to prevent hypoglycaemia and severe hyperglycaemia (10). Guidance to emergency/admissions departments should include glucose measurement on all admissions, as a significant number of COVID-19 positive patients not previously known to have diabetes present with marked hyperglycaemia. Additionally, ketones should be checked both in everyone with known diabetes and in those without known diabetes who present with a blood glucose above 12 mmol/L. Anecdotal reports suggest that unusual presentations of diabetic metabolic emergencies including diabetic ketoacidosis (DKA) or mixed DKA and Hyperosmolar Hyperglycaemic State (HHS) in type 2 diabetes with the risk of DKA being greater in those on SGLT-2 inhibitors. SGLT-2 inhibitors and metformin should therefore be stopped in all patients on acute presentation, given potential association with metabolic emergencies and of AKI.. Figure 1 gives useful ‘Front Door’ guidance for individual patients based on experience from UK centres (https://www.diabetes.org.uk/resources-s3/public/2020-04/COvID_Front_Door_v1.0.pdf). Non-COVID-19 related DKA and HHS should be managed using standard protocols and additional support implemented to reduce admissions in known high-risk individuals (see subsequently).

## Diabetes-related population health risks during the COVID-19 pandemic

In the general population, restrictions on travel and person-to-person contact may lead to worsening of risk factors for complications and poorer health outcomes from established diabetes and rising diabetes incidence through:

### Changes in lifestyle

- More sedentary behaviour/less activity
- Lack of access to healthy foods
- Lack of face-to-face, peer and professional support for weight loss/healthy lifestyle
- Reduced carer/family support for self-management
- Increased alcohol consumption (11)
- Deterioration in mental health, due to stress and isolation and reduced wider family network/peer support.

### Reduced population complications screening/acute treatment changes

- Failure to monitor renal function appropriately in people with CKD leading to avoidable admissions with fluid overload, anaemia or electrolyte disturbance
- Failure to monitor blood pressure/other CVD risk factors appropriately leading to preventable CVD events
- Delay in management of diabetes-related foot ulcers/foot infections and sight-threatening retinopathy leading to preventable amputations and blindness.
- Inappropriate continuation and discontinuation of medications (12)

## Role of diabetes specialist/community teams during the time of COVID-19

It is vital to *maintain patient safety* while accelerating patient flow through the hospital and delivering closely managed outpatient services to prevent avoidable admissions and readmissions. Achieving this involves:

1. **Reducing risk of COVID-19 infection** through clear articulation of evolving COVID-19 advice to people with diabetes, to enable understanding of risk status and expectations around healthcare service interaction
2. **Preventing** people with diabetes in the community falling ill from diabetes-related complications (hypoglycaemia, diabetic ketoacidosis (DKA), hyperosmolar hyperglycaemic state (HHS), and foot infections)
3. **Assisting** people with diabetes out of hospital when they become unwell to prevent admission for diabetes-related complications (as mentioned previously)
4. **Supporting inpatient teams** (especially on COVID wards) to manage people with acute diabetes complications safely, including those in ICU with high insulin requirements
5. **Providing education for frontline inpatient teams** who are unfamiliar with diabetes management
6. **Facilitating early discharge** to the community with programmed daily diabetes follow-up to prevent readmission
7. **Supporting primary care** diabetes management

It is critical to maintain a skeleton service capable of delivering 1, 2, 3 and 6 to keep people with diabetes out of hospital, as well as a more significant service for diabetes care in hospital. This should ideally include a limited inpatient/community weekend service.

## Practical advice for ongoing out-patient management

Challenges of outpatient care delivery are compounded by reduced staffing levels due to illness and deployment of clinicians to ‘frontline’ duties. Smaller numbers of staff are thus manning skeleton outpatient services. The duration of outpatient service disruption is currently uncertain.

### (1) Short-term service interruption guidance (e.g. 1–3 months)

#### Suggestions:

- Face-to-face clinic review should only occur where health benefits of attendance outweigh the risks associated with patient movement (i.e. potential individual and wider societal COVID-19 spread)
- Pregnancy, foot services, and management of newly diagnosed people with Type 1 diabetes may need to continue at full capacity, as per national guidance (13)
- Delay routine screening tests unless the patient is at very high risk of deterioration, for example, due to age, trajectory of previous test results or past history
- Accept that numbers achieving routine care processes (e.g. BP, lipid, HbA1C, renal function, ACR, feet and eye screening (14)) will reduce
- Continue to advocate national/personal targets for metabolic parameters, using remote monitoring where possible
- Aim for complications prevention through a focus on highest risk populations
- Become familiar with and signpost patients to credible online sources of advice, education/self-management and remote monitoring tools
- Pool resources across regions to resource diabetes care services optimally
- If staff resource depletes due to illness/reallocation to COVID-19 duties, then focus on supporting highest risk patients only
- Implement and publicise a telephone/online advice service

**Figure 2:**
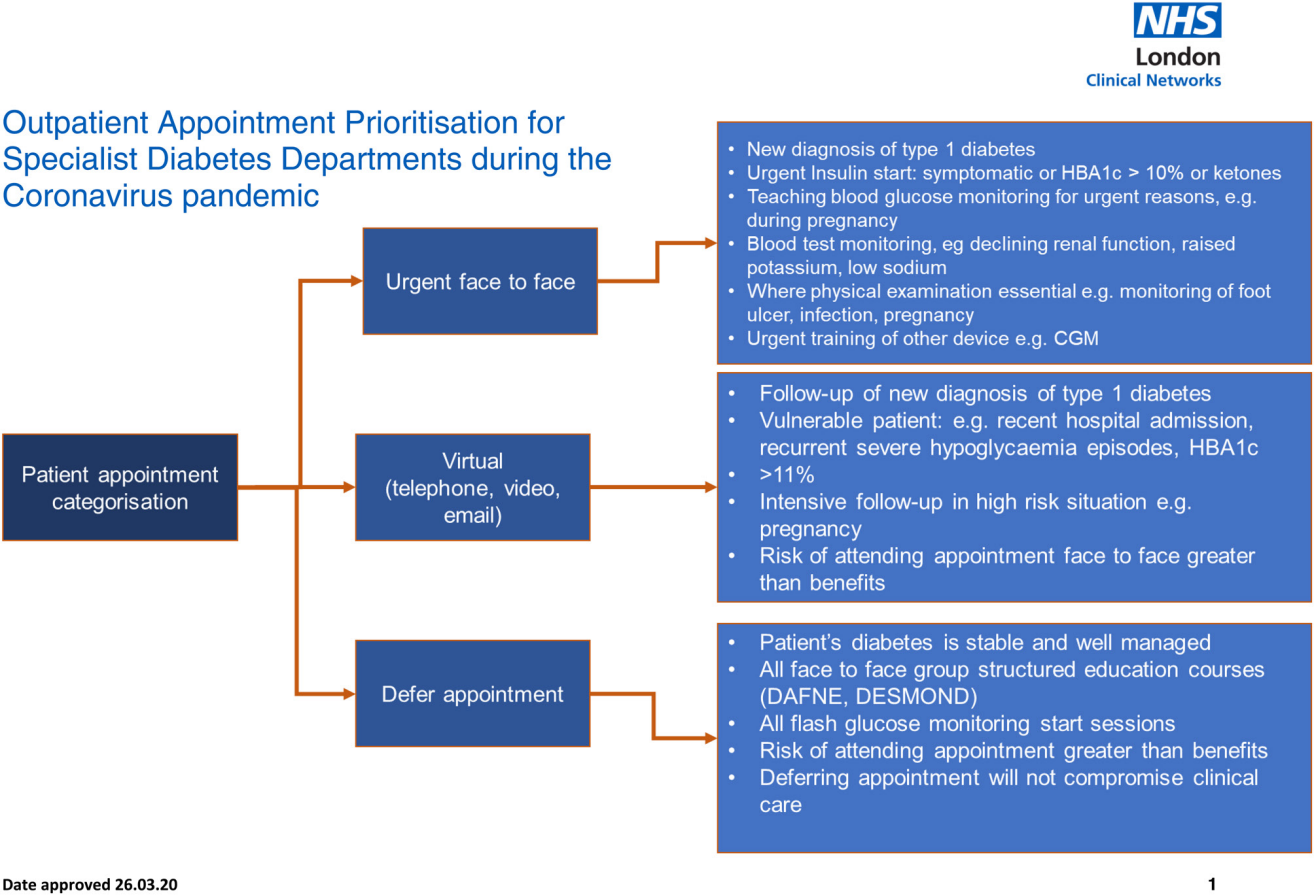
Represents a consensus flow chart produced by the NHS London Clinical Network for Outpatient Appointment Prioritisation for Specialist Diabetes Departments during the Coronavirus. Included with permission from Stephen Thomas as group lead.

Figure 2 (15) contains a suggested outpatient prioritisation flow chart.

### (2) Medium to longer term service interruption (e.g. 3–12 months)

As normal service interruption lengthens, the risk of harm due to delayed screening that could have informed early intervention and complication avoidance increases. It may become necessary to enable routine diabetes screening, using facilities and procedures that minimise COVID-19 transmission risk. Patients at highest risk of deterioration should be prioritised using risk algorithms if possible.

## Risk assessment in care delivery

While some IT-integrated risk assessment tools are available (e.g. Eclipse; https://www.prescribingservices.org), they have not been adapted for risk assessment around routine care delivery during COVID-19, which is generally being done intuitively. More advanced data-driven machine learning models could support decisions. Models already exist for prediction of (1) mortality (16), (2) glycaemic control deterioration, (3) DKA (17), (4) eye disease (18, 19, 20), (5) foot ulcers/amputations, (6) kidney failure (20) and (7) cardiovascular complications (20). Most models, however, remain within academic papers and few are linked to front-line user interfaces supporting real-time care prioritisation (21). In addition, previously developed models were trained in data drawn from contexts where populations studied were largely attending regular, scheduled clinical review/screening visits. The current pandemic has driven an unprecedented degree of routine clinical review deferral. An excess all-cause mortality in weeks 12 and 13 of 2020 has been observed when compared with the same weeks in 5 years to 2019, which is not fully attributable to COVID-19 (22), suggesting that COVID-19 may be impacting healthcare system performance. Machine learning models predicting individual risk of adverse outcomes due explicitly to COVID-19 service disruption could prove useful for risk stratification and care planning. We support a call for urgent work in this area.

## Outpatient care delivery; potential for remote/digital tools

1. **Consultations**
  In compliance with the ‘stay at home’ mandate, most structured education and non-urgent routine care has been cancelled or is being delivered where necessary through remote consultation. Remote consultations may take longer than face to face, but rates of ‘nonattendance’ may be lower and patient satisfaction can be high (23). Online prescribing, reordering, dispensing and delivery should be encouraged.
2. **Education**
  Patient self-management is key to good diabetes outcomes, encompassing management of lifestyle, insulin dose titration, foot self-care, and adherence to treatment plans. Healthcare professional (HCP) support can be delivered using online tools or telephone, although many patients value the use of physical aids and peer support of face-to-face/group sessions. Online structured education resources include: online DESMOND (https://www.desmond-project.org.uk), BERTIE (type 1 diabetes) (https://www.bertieonline.org.uk), and the MyDiabetesMyWay (MDMW)/MyWay Digital platform (https://mywaydigitalhealth.co.uk). Apps incorporating 1:1 digital coaching include: Oviva (https://oviva.com/uk/en/), Changing health (https://www.changinghealth.com), Our Path/Second Nature (https://www.changinghealth.com), Liva (https://livahealthcare.com), Omada (https://www.omadahealth.com) and Livongo (https://www2.livongo.com). Some providers are offering discounted or free services during the pandemic. Use of public information sites for COVID-19/sick day rules are valued. The NHS Scotland MyDiabetesMyWay COVID-19/sick day advice page had 13 443 views with 98% positive ratings/comments (*n*=368) over 3 weeks since 16 March. Digitally supported diabetes self-management has the potential to be effective (24) and could be cost saving (25). Whether uptake increases during the pandemic period, preventing care deterioration, remains to be seen (26).
3. **Remote monitoring**
  1. **Glucose monitoring**: Remote upload of home glucose recordings enabling remote healthcare professional review and feedback is possible through proprietary systems such as LibreView, CareLink, Clarity, glucose data aggregator systems such as Diasend and Tidepool, and other independent applications. Pre-COVID-19, the number of ‘at home’ glucose uploads vs ‘in-clinic’ uploads was limited. Early NHS Scotland data (**Personal Communication) suggest a steady (7%) increase in home LibreView uploads during last 4 weeks. Patients with gestational diabetes (GDM) attend hospital approximately fortnightly during later pregnancy. Glucose data feedback could be facilitated remotely using technology including specific systems, for example, Sensyne Health’s GDM-Health app (https://www.sensynehealth.com/gdm-health).
  2. **Activity, physiological parameters and ‘internet of things’ monitoring**: Many apps/online platforms enable sharing of home activity, blood pressure, weight and other readings with healthcare teams, either through automatic/bluetooth connectivity or manual data entry (e.g. https://mwdh.co.uk, https://mymhealth.com). These could support ongoing lifestyle change and enable treatment optimisation.
  3. **Biochemical blood and urine testing**: Nationally recommended blood and urine screening (13) may need deferred. Remote consultation without recent HbA1c results is challenging, particularly if no home glucose data is available. Products enabling HbA1c home testing (27, 28), and systems using smart phone embedded technology enabling ‘at home’ diagnostics (e.g. DipiO; https://healthy.io/Testcard; https://testcard.com for urine Albumin Creatinine Ratio), could help, but none have been widely implemented to date.
  4. **Foot care**: Remote solutions to support neuro-vascular assessment, preventative podiatry work and active foot disease treatments are limited, but simple at home neuropathy tests such as the ‘Ipswich touch the toes test’ may be sufficiently reliable when performed by a relative or carer (29), and home foot pressure mats/remote neuropathy detection systems could assist where available (30). Home upload of digital photographs with or without additional wound tracking applications (e.g. https://healthy.io/wound) may reduce attendance episodes for ulcer treatment.
  5. **Eye screening**: Currently, eye screening is widely facilitated through industrial screening cameras in clinical centres linked to systematic image review with or without artificial intelligence grading (31). Smart phones have been used as retinal cameras, but the technology does not yet enable individual home ownership and is currently largely utilised through community hubs, for example, in rural India (32).

As routine complication screening declines, a deterioration in outcomes is predicted. Whether remote solutions can be rapidly implemented to plug the gap remains to be seen. Diabetes screening, monitoring and education will ultimately be deliverable from the home, through standard personal mobile devices. The main barriers will be changing healthcare organisations, procurement/reimbursement practices, and supporting end users. While 80% of UK adults own smartphones (33) and 95% between the age 16 and 74 in UK access the internet regularly (34), the majority of people with type 2 diabetes are over the age of 65 (35, 36) and some may lack skills to independently use digital tools. Technology user support in healthcare is distinctly under-resourced, and changes in service delivery during this stage of the pandemic may increase health inequalities. There are also concerns that mental health may deteriorate due to stress and social isolation. Online tools and telephone support may require signposting for high-risk individuals.

## Innovation and procurement/commissioning

The COVID-19 pandemic has enforced a period of disruptive innovation. As a result, information governance barriers are crumbling and procurement rules are being rewritten. One NHS trust saw an 18-month planned Microsoft Teams implementation happen over a weekend and a 20-year rule disallowing healthcare professional-patient email contact changed overnight. In the USA, reimbursement barriers for telemedicine services are rapidly evaporating. A reform of procurement procedures has enabled rapid commissioning and deployment of services and systems. The first wave has rightly focused on solutions with immediate impact, such as COVID-19 testing kits, vaccine development, hand wash, and ventilators, but attention may turn to tools supporting chronic conditions management if social distancing measures continue.

## Diabetes opportunities resulting from COVID-19 restrictions

- The use of technology/remote consultations may increase in the long term, meaning more flexible care delivery accommodating patient lifestyle, work and carer commitments (with secondary environmental (less travel), and economic (less time off work) benefits (37)), replacing rigidly timed protocolised face-to-face appointments. Care delivery may also better support acute needs, including proactive delivery of sick day guidance.
- Rigid reliance on standard guidelines for populations may give way to more individualised patient-centred care. Risk stratification may increasingly become part of service delivery, with proportionately more time focusing and re-engaging those at highest risk of deterioration including disengaged populations. This could transform care outcomes and cost of delivery; currently a small percentage of high-risk diabetes patients consume disproportionate costs due to treatment of complications (38, 39).
- Efficiency in care delivery could improve through continuation of COVID-19 clinic service and personnel restructuring.

## Conclusions

The COVID-19 pandemic has required rapid adaptation of care delivery, supported by governmental and national body recommendations, but has created a conflict around where care priorities should lie. Whether similar systematic change is possible in less developed, lower resourced countries remains to be seen. People with diabetes could suffer disproportionately during COVID-19. Service restructuring and digital tools may reduce risks of health decline during this period. While the current focus is on limiting the terrible harm caused by the pandemic, it is possible that COVID-19 might leave a legacy of accelerated deployment of innovative pathways and approaches to chronic disease management supporting person-centred care.

## Disclaimer

Due to the emerging nature of the COVID-19 crisis, this document is not based on extensive systematic review or meta-analysis, but on rapid expert consensus. The document should be considered as guidance only; it is not intended to determine an absolute standard of medical care. Healthcare staff need to consider individual circumstances when devising the management plan for a specific patient.

## Declaration of interest

Debbie Wake is Co-founder and CEO of MyWayDigital Health. David C. Klonoff is a consultant to Abbott, Ascensia, Dexcom, EOFlow, Fractyl, Lifecare, Novo, Roche, and Thirdway. Robert Semple is Deputy Editor of the *European Journal of Endocrinology*. He was not involved in the editorial or review process of this paper, on which he is listed as an author.

## Funding

RS is supported by the Wellcome Trust http://dx.doi.org/10.13039/100010269, grant 210752/Z/18/Z.
